# Clinical Validation of the Champagne Algorithm for Epilepsy Spike Localization

**DOI:** 10.3389/fnhum.2021.642819

**Published:** 2021-05-20

**Authors:** Chang Cai, Jessie Chen, Anne M. Findlay, Danielle Mizuiri, Kensuke Sekihara, Heidi E. Kirsch, Srikantan S. Nagarajan

**Affiliations:** ^1^National Engineering Research Center for E-Learning, Central China Normal University, Wuhan, China; ^2^Department of Radiology and Biomedical Imaging, University of California, San Francisco, San Francisco, CA, United States; ^3^Department of Advanced Technology in Medicine, Tokyo Medical and Dental University, Tokyo, Japan; ^4^Signal Analysis Inc., Hachioji, Japan; ^5^Department of Neurology, University of California, San Francisco, San Francisco, CA, United States

**Keywords:** source localization, epilepsy, source imaging analysis, magnetoencephalography, spike analysis, brain source imaging, brain source localization

## Abstract

Magnetoencephalography (MEG) is increasingly used for presurgical planning in people with medically refractory focal epilepsy. Localization of interictal epileptiform activity, a surrogate for the seizure onset zone whose removal may prevent seizures, is challenging and depends on the use of multiple complementary techniques. Accurate and reliable localization of epileptiform activity from spontaneous MEG data has been an elusive goal. One approach toward this goal is to use a novel Bayesian inference algorithm—the Champagne algorithm with noise learning—which has shown tremendous success in source reconstruction, especially for focal brain sources. In this study, we localized sources of manually identified MEG spikes using the Champagne algorithm in a cohort of 16 patients with medically refractory epilepsy collected in two consecutive series. To evaluate the reliability of this approach, we compared the performance to equivalent current dipole (ECD) modeling, a conventional source localization technique that is commonly used in clinical practice. Results suggest that Champagne may be a robust, automated, alternative to manual parametric dipole fitting methods for localization of interictal MEG spikes, in addition to its previously described clinical and research applications.

## 1. Introduction

Epilepsy is a neurological disorder that poses an important challenge due to its prevalence, as it affects about 0.8–1% of the world population (Organization et al., 2005; Moshé et al., 2015). While typical pharmacological treatments work to control seizures for the majority of people with epilepsy, roughly one-third suffer from drug-resistant epilepsy (Lamberink et al., 2017), leaving surgery as a possible option for treatment (Ryvlin et al., 2014) for those in whom the seizure onset zone (SOZ), where seizures arise, can be identified (Zijlmans et al., 2019). Magnetoencephalography (MEG) is increasingly used for presurgical planning in people with focal onset epilepsy refractory to pharmacotherapy (Koster et al., 2020). MEG uses recordings of minute extra-cranial magnetic fields produced by cortical activity, and is thereby able to display changes in brain state with a time resolution below 1 ms. Sources of such activity can be localized with an accuracy of several millimeters (Wheless et al., 2004). Compared to other existing techniques, MEG has the advantage of being completely non-invasive, and a wide range of high-resolution analysis techniques may be applied to broadband MEG datasets.

Like EEG, MEG can record both ictal and interictal data but because patients are commonly studied in the MEG lab as outpatients for only a limited amount of time, it is relatively rare to capture a seizure, and most commonly, interictal epileptiform discharges (spikes and sharp waves) are captured and source modeling is carried out. The most widely used technique for localizing interictal epileptiform discharges is a two-step process: (1) visual detection and identification of spikes in the raw MEG. followed by (2) calculation of the location and orientation of equivalent current dipoles (ECD) that best account for the observed magnetic field and review of the resulting fits (Vrba and Robinson, 2001; Wheless et al., 2004). This method has been proven to provide reliable and useful information for presurgical planning by several studies (RamachandranNair et al., 2007) that also concluded that invasive intracranial recording techniques might eventually be replaced by MEG (Knowlton et al., 2006). Such procedures require manual review of ECD results, which can be time-consuming, requires trained and experienced scorers, and contains a considerable amount of subjectivity in the results. Additionally, users of ECD methods typically attempt to represent activity at only one time point associated with a spike, and so such models are inherently limited in their ability to capture activity that represents the entire time course of such activity.

As a complement to ECD methods, our lab developed and uses a robust brain source imaging algorithm, Champagne, derived based on an empirical Bayesian inference and incorporating deep theoretical ideas about sparse-source recovery from noisy, constrained measurements. Champagne improves source reconstruction performance evaluated using reconstruction accuracy, robustness, and computational efficiency measures (Wipf et al., 2010). Experiments with simulated and real data have shown that Champagne is robust for multiple complex brain sources even under high interference from correlated sources or with noisy data (Owen et al., 2012). The latest extension of the Champagne algorithm incorporating noise learning is able to reconstruct complex focal source activity when corrupted by high levels of noise and interference, while maintaining its typical high performance features (Cai et al., 2020), without the need for baseline or control data. Champagne with noise learning is thus especially suitable for uses like the localization of interictal epileptiform spikes, where there is a focal brain source and high levels of noise may co-exist.

Here, we evaluate a source localization pipeline for analysis of manually identified epileptiform discharges using the Champagne algorithm with noise learning. Once selected, this pipeline provides a fully automated reconstruction of the source of these interictal events. Our study demonstrates the validity of this approach in a cohort of 16 patients with focal epilepsy and compares it with an established clinical source localization technique—parametric dipole fitting—to assess its practicality and utility.

## 2. Materials and Methods

### 2.1. Patients

In this paper, we chose 16 patients with medically refractory focal epilepsy, one consecutive group from 2015 and one consecutive group from 2019, who had MEG at the UCSF Biomagnetic Imaging Laboratory and met the following inclusion criteria: all patients were referred for MEG at UCSF as part of pre-surgical planning. UCSF Committee on Human Research approved all procedures. All study procedures were conducted based on the Declaration of Helsinki. Since many patients were either external referrals or recently scanned, therefore long-term outcome data was not available for them. Participants gave their written informed consent to participate in the study, or in some cases the need for consent was waived for the purpose of retrospective analysis by the UCSF Committee on Human Research.

### 2.2. Structural Images

All patients had magnetic resonance imaging (MRI) performed at 1.5 or 3 T that was used for visualization of source localization results. This study used the protocol including the following sequences: (1) a T1-weighted, 3D spoiled gradient-recalled echo in a steady state sequence with TR 34 ms, TE 3–8 ms, flip angle 30°; (2) a T2-weighted 3D fast-spin echo sequence with TR 3000 ms, TE 105 ms. Both sequences had the following detail features: slice thickness of 1.5 mm, matrix 256 × 256 × (108−140), and a field view of 260 × 260 mm with skin-to-skin coverage including the nasion and preauricular points.

### 2.3. Recordings

All MEG recordings were collected when the participants were lying with their eyes closed in a magnetically shielded room. Between 20 and 60 min of continuous, interictal, resting state MEG were recorded with a 275 channel whole-head CTF Omega 2000 system (VSM MedTech, Coquitlam, BC, Canada), using a passband of DC to 70 Hz and a sampling rate of 1,200 Hz, then downsampling to 600 Hz. In addition, concurrent scalp electroencephalographic (EEG) data were recorded using 19 leads placed according to the international 10–20 system, along with an electrocardiogram channel and eye channels to assist in the identification of artifacts. Some patients were sleep deprived and reached various stages of sleep during the recordings.

### 2.4. Signal Analyses

A single-sphere (for ECD) and a multi-sphere (for Champagne algorithm) head model were created for each patient, based on the structural MRI images. Co-alignment of structural and functional images was achieved by marking three prominent anatomical points (nasion and preauricular points) of the subject's head on the MRI images and localizing three magnetic fiducials attached to the same points. All recordings were reviewed by experienced MEG technologists (MM, AF, DM) and clinical neurophysiologists and epileptologists (HEK, HC), and the peak of all epileptiform spikes was marked manually based on both the MEG and EEG recordings. In addition to the continuous recordings, spike-locked datasets were created starting 1 s before and ending 1 s after each spike and corrected for baseline-offset using the first 700 ms. Spikes <1 s after a previous spike were excluded. An equivalent current dipole model was fitted to the magnetic field recorded with the entire MEG sensor array during each single spike (ECDss). For each spike, the sampling point was used that yielded the model with the smallest residual variance. As a general rule, spikes with dipoles having >10% residual variance (aka error) were rejected. Spike topography, moment strength, and orientation were also considered for selection of ECDss. All source localizations were done using software provided by VSM MedTech (Coquitlam, BC, Canada). For the pipeline based on the Champagne algorithm proposed here, we first remove the DC offset and band-pass filter the time course for each subject with at 1–45 Hz, then we set the time window for each spike as 25 ms before and 75 ms after the spike peak. To run more complex spike localizations, we also concatenate all spikes then reconstruct the brain sources by running Champagne directly.

### 2.5. Performance Validation

First, we evaluate the performance of Champagne for 14 “straightforward” cases, defined as those having spikes with localization error <10% using ECDss. Hit rate and *A*′ values are derived using FROC analysis as previously described (Owen et al., 2012; Sekihara, 2016; Cai et al., 2018, 2019).

Since the Champagne algorithm is a focal and sparse distributed reconstruction algorithm that estimates source amplitude for all voxels in the brain, to evaluate algorithm performance we use robust thresholding methods as found in prior simulations (Cai et al., 2020). We define a successful localization as one that meets the following criteria: we identify active sources estimated as those brain sources whose amplitude exceeds 1% of the largest amplitude of the estimation. This threshold ensures that only significant estimated sources were selected. Amongst all estimated sources, success of a localization requires that there should be at least one estimated source location within 2 cm of the limits of the source location estimated by ECDss (with localization error <10%).

Second, we evaluate the performance of Champagne algorithm for spikes where data were “not straightforward.” For this, we looked at two patients who had some spikes that could be modeled with ECDss with acceptably low error (<10%) to a consistent cluster of plausible sources that also matched clinical data (eg seizure semiology, ictal EEG), but ALSO had other spikes with similar waveforms in sensor space (and on simultaneous EEG) but with errors >25% when modeled using ECDss. We defined “ground truth” in this scenario as the average of the coordinates of the ECDss localization that had errors <10%. For the spikes with errors >25% by ECDss, we applied the Champagne algorithm to generate source models. Then we compared the Euclidean distance between the ground truth and the location of these “high-error spikes” (i.e., those with ECDss localization error >25%) as estimated by ECDss and then by Champagne.

Finally, we evaluated the performance of the Champagne algorithm for the localization of more complex spikes. Here, we ran Champagne on data from one patient with spikes localizing to two clusters of ECDss locations. We first concatenated all spikes, then localized this interictal epileptiform activity using Champagne.

Please see Supplementary Table 1 for clinical information including MRI, PET, SPECT, and video-EEG results.

## 3. Results

**Figure 1:**
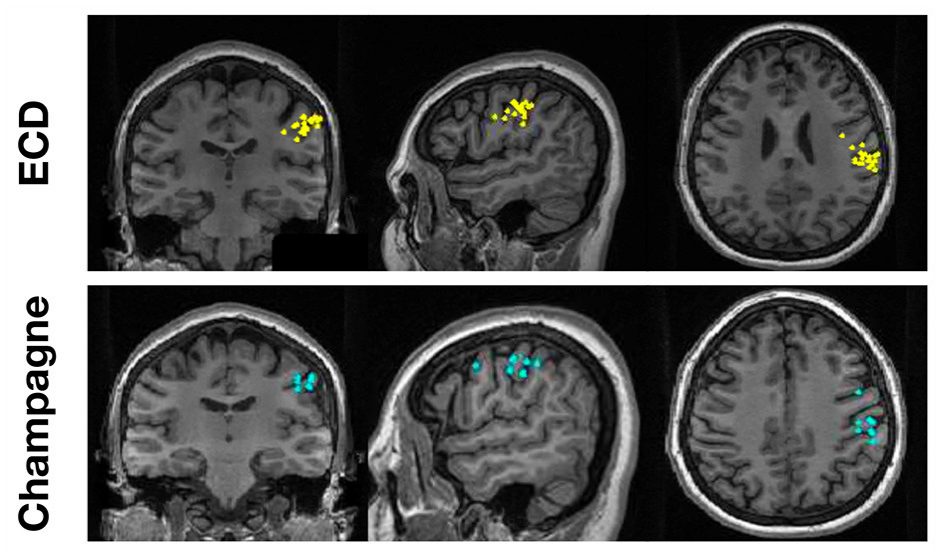
A representative “straightforward” case comparing ECDss and Champagne algorithms for spike localization (Subject 13). The top row shows ECDss fits with errors <10% (yellow dots); the bottom row shows Champagne localization for the same spikes (blue dots).

To evaluate the performance of Champagne for “straightforward” cases, we chose spikes with ECDss localization errors <10% from 14 people with intractable epilepsy. Figure 1 shows these spike localization using ECDss, and localizations for the same spikes using the Champagne algorithm, for a representative case. As is shown, Champagne with noise learning yields similar results.

**Figure 2:**
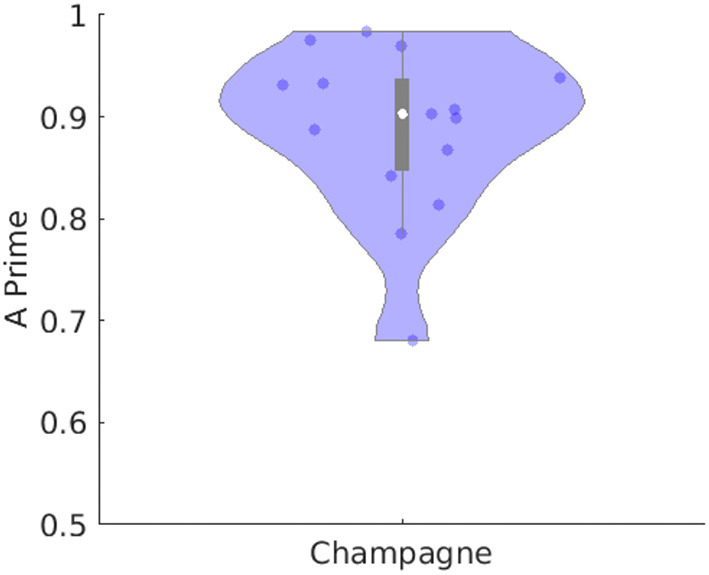
Performance of Champagne for 14 subjects with spikes where ECDss error <10%. The violin plot shows the distribution of *A*′ metric across all subjects; each dot represents data from one person. Note that the white point represents the mean, the box subtends the middle quartiles and the whiskers the outer deciles.

Table 1 lists number of spikes recorded for each of the 14 subjects in the “straightforward” group, with the ability of Champagne as compared to ECDss to localize the same spikes in 14 subjects, including hit rate and *A*′ metric calculated as described previously (Owen et al., 2012; Sekihara, 2016; Cai et al., 2018, 2019). Figure 2 illustrates this performance of Champagne across this group of 14 people with intractable epilepsy. Note that the mean *A*′ metric across all 14 subjects is 0.9, suggesting that the localization provided by the Champagne algorithm is similar to that of ECDss (The *A*′ metric is >0.75% in one subject because we recorded only five spikes during this study).

**Table 1:** Overview of source localizations of interictal epileptiform activity recorded from 14 patients.

**ID**	**1**	**2**	**3**	**4**	**5**	**6**	**7**	**8**	**9**	**10**	**11**	**12**	**13**	**14**
Num of spikes	36	18	21	5	6	27	9	25	16	10	15	11	21	22
Hit ratio	0.6111	0.7222	0.8571	0.4	0.6667	0.8889	0.7778	0.84	0.9375	1	1	0.9091	1	0.8636
A′ metric	0.7855	0.8416	0.9072	0.6813	0.8142	0.9326	0.8675	0.8987	0.9317	0.9746	0.9694	0.9383	0.9834	0.9032

**Figure 3:**
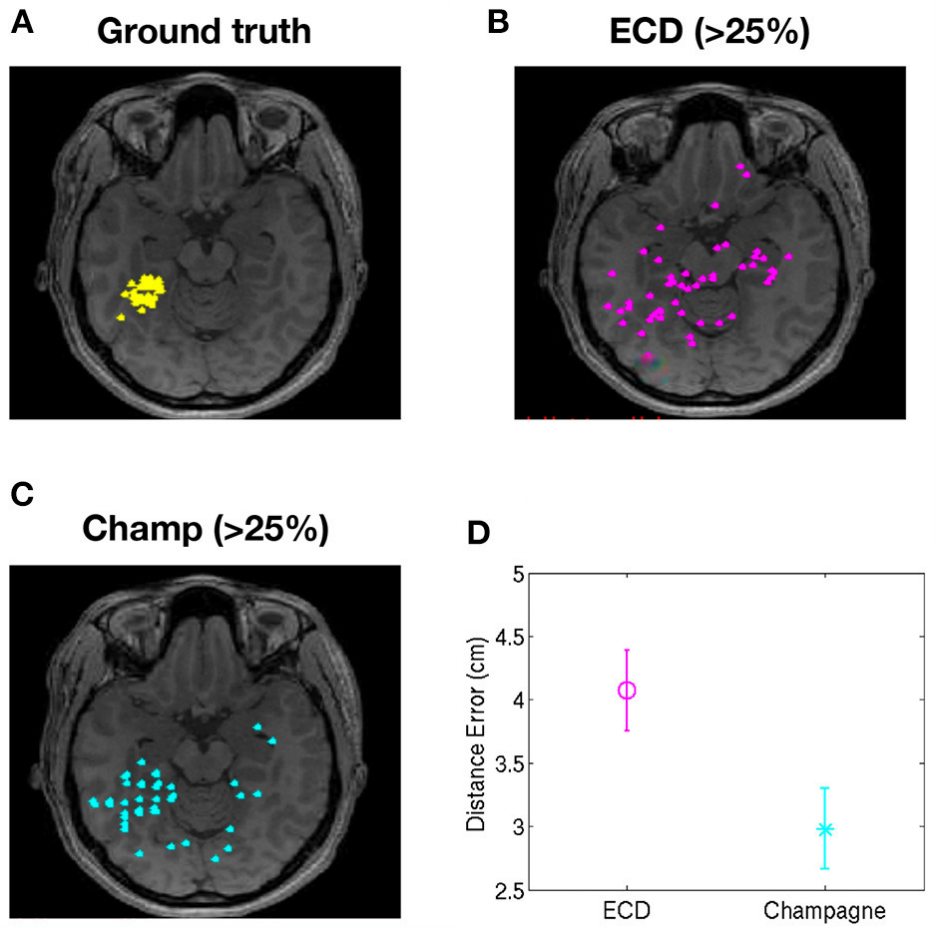
Localization of high-error spikes by ECDss and Champagne algorithms: Subject 1. **(A)** Ground truth as defined by localizations of spikes by ECDss with errors <10%. **(B)** Localizations of spikes by ECDss with error >25% **(C)** Localization results when spikes with error >25% by ECDss were then localized using the Champagne algorithm. **(D)** Distance between the ground truth **(A)** and the localization result by ECDss **(B)** and Champagne **(C)** for those spikes with ECDss error >25%. For **(D)**, whiskers indicate SEM.

**Figure 4:**
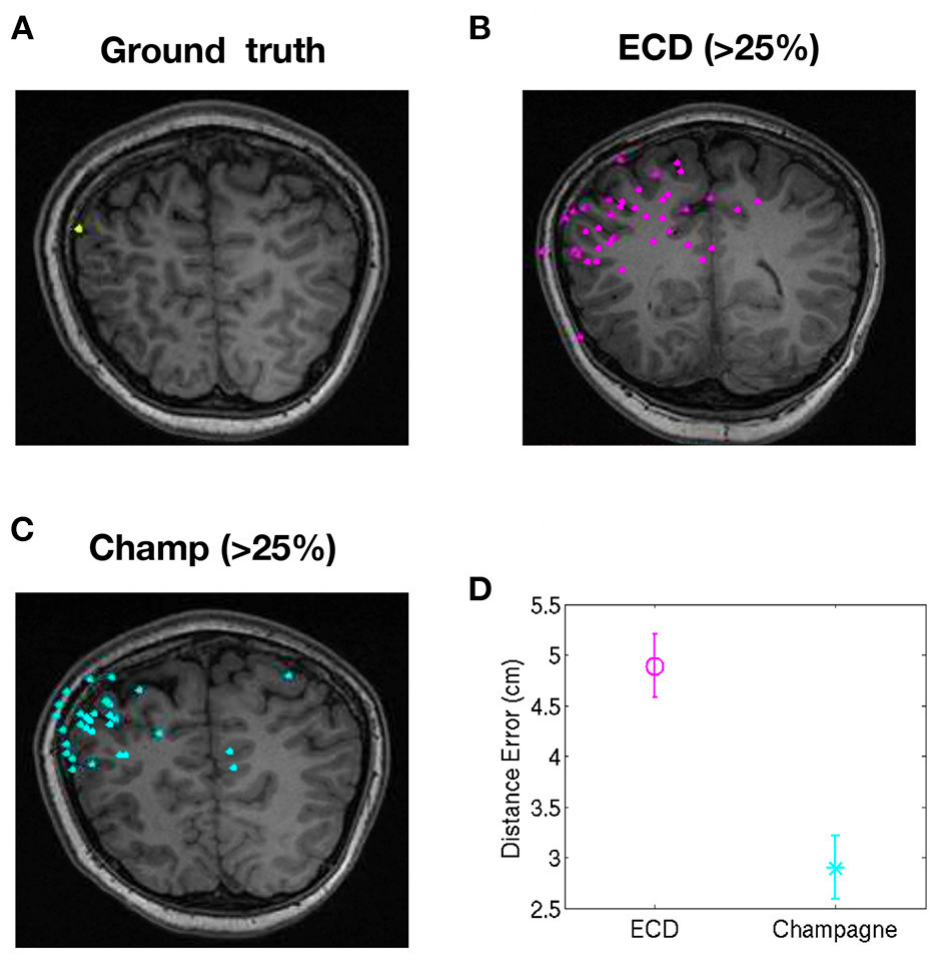
Localization of high-error spikes by ECDss and Champagne algorithms: Subject 15. **(A)** Ground truth as defined by localizations of spikes by ECDss with errors <10%. **(B)** Localizations of spikes by ECDss with error >25% **(C)** Localization results when spikes with error >25% by ECDss were then localized using the Champagne algorithm. **(D)** Distance between the ground truth **(A)** and the localization result by ECDss **(B)** and Champagne **(C)** for those spikes with ECDss error >25%. For **(D)**, whiskers indicate SEM.

To evaluate the performance of our newer pipeline for spikes that are “not straightforward,” we ran the Champagne algorithm on spikes where ECDss fitting had yielded errors >25% in data from two patients (NB one of these two patients had several distinct spike populations/morphologies, one of which was described, analyzed and included in the previous group of 14 “straightforward”). Note that these were spikes where the morphology on EEG and MEG suggested that all spikes (those that modeled with higher vs. lower error using ECDss) might arise from the same source. Here, we defined as ground truth the set of source localizations with low error (<10%) using ECDss on the majority of spikes from the same patients. Figure 3 shows the localization result of more than 49 “not-straightforward” spikes using ECDss and Champagne algorithms (A). The localization results of ECDss with error >25% are distributed over whole brain (B), while the localization results of Champagne are more focal (C). Distance between the ground truth and the ECDss result is 4 cm, much higher than that of Champagne 3 cm. Figure 4 shows similar data for another patient with 38 spikes (errors >25%).

**Figure 5:**
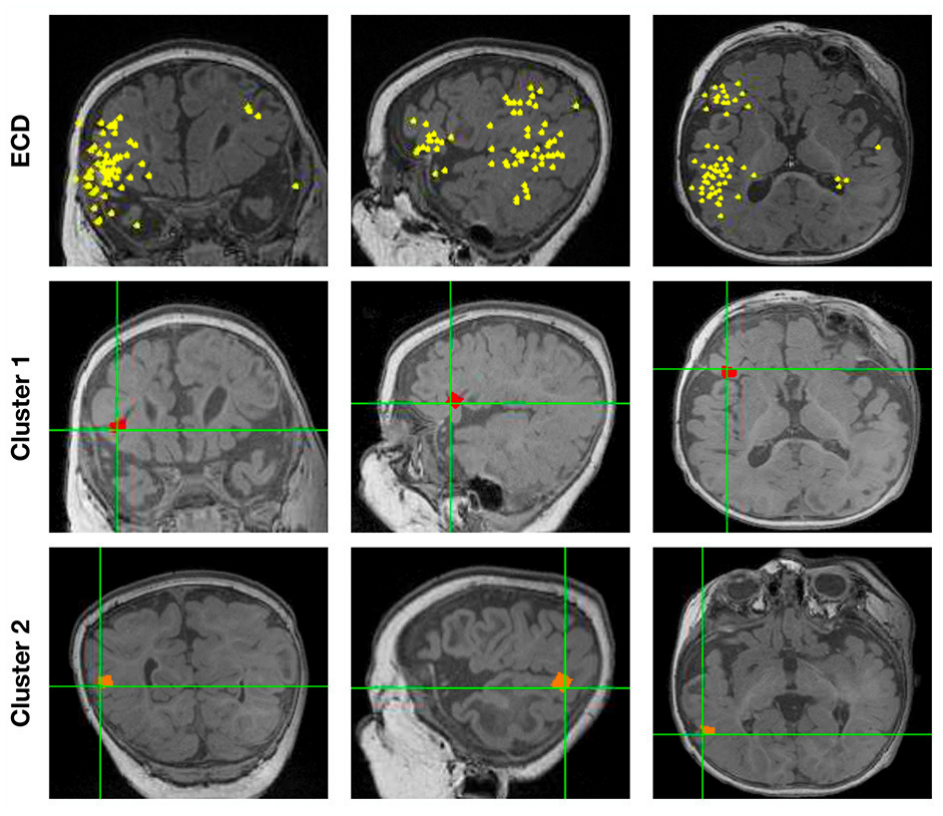
The localization results for one patient (Subject 16) with spikes in two clusters by ECDss and by Champagne algorithm.

Figure 5 shows the localization results of one more complex scenario, when spikes localize to clusters of dipoles. Here, we first ran ECDss in an additional patient. We show the localization results in the first row of Figure 5. We then concatenated all spikes and localized them using Champagne. As shown from the first row of Figure 5, with ECDss localizations are classified into two clusters. The second and third rows present the Champagne localization results. We can see that Champagne is able to localize these two clusters of interictal epileptiform activity.

## 4. Discussion

Over the past three decades, whole-head MEG has continued to prove its value as an important tool for the evaluation of people with medically refractory epilepsy. As a key part of the presurgical workup, it can assist in the identification of the SOZ by providing corroboration of hypotheses and enabling resection or implantation of electrodes for staged evaluations. MEG interictal epileptiform activity is one of many potential surrogates for SOZ, and though emerging MEG biomarkers including high frequency oscillations may supplant its role (Frauscher et al., 2017), it is the most well-established, familiar, and widely used. The standard method of source localization is the localization in individual interictal epileptic spikes using dipole fitting (ECDss) (Bagic et al., 2011). However, this technique is operator-dependent and time-consuming. Dipole fitting procedures often do fail under both low and high SNR regimes, and when spikes do not show a clear dipolar topography, and has shown to have weak localization accuracy (Kobayashi et al., 2005; Fujiwara et al., 2012).

To explore the performance of Champagne algorithm with noise learning for clinical use in the localization of interictal epileptiform discharges—“spikes”—we compared its performance against ECDss in three common scenarios.

First, for “straightforward” cases, where ECDss solutions had low error, Champagne with noise learning yields similar results and the mean *A*′ metric across all 14 subjects is around 0.9. This illustrates that Champagne can perform equivalently to ECDss in this common use scenario.

We then address a second common scenario: where there spikes where ECDss fails due to high error. To test this we looked specifically at a group of people who had a population of spikes that could be modeled using ECDss to yield a cluster of dipoles that were then used as “ground truth.” They also had spikes that were more difficult to model with ECDss (albeit having a similar waveform in sensor space and similar EEG morphology to those with lower error ECDss models). When these were modeled, localization results using Champagne were more focal than were results generated by ECDss. In addition, Champagne localizations were much closer to the low error “ground truth” ECDss localizations than were the high error ECDss fits. In these cases, therefore, we show how we can use Champagne to salvage spikes that could not be confidently modeled using ECDss due to high error.

We also address a third scenario, in one patient with two plausible clusters of ECDss sources corresponding to interictal spikes. For this patient, the Champagne algorithm is able to resolve these two clusters and to reconstruct independent sources.

The pipeline we describe here does require manual detection and identification of interictal epileptiform activity at an early analysis stage. Later stages of the process are relatively automated, and can be used by operators with variable degrees of neurophysiology expertise. In recent years, similar Bayesian iterative approaches have been applied to this problem in order to develop semi-automated algorithms for clinical use. For example, an automated multidipole iterative Monte Carlo approach called “SESAME” was evaluated by Luria et al. (2020) and was concordant with standard ECD fitting done by experienced staff. Pellegrino et al. (2018) applied the coherent Maximum Entropy of the Mean (cMEM) to yield distributed magnetic source images (dMSI) that provided improved accuracy compared to ECD, also having the advantage of user-independent results; as dMSI results provide spatial and temporal voxelwise information, they can offer information about anatomic extent of source beyond that available from ECD. We note that our approach is comparable to these similar approaches that others have taken, and performance compared to standard ECD methods is likewise comparable. Together, these experiences suggest clear and robust alternatives to parametric dipole fitting procedures.

## 5. Conclusion

The application of the Champagne algorithm with noise learning allows the localization of underlying brain activity in a precise and objective manner without the need for additional “baseline” or “control” data to estimate contributions to sensors from noise. Here, we demonstrate our use of the Champagne algorithm in some scenarios commonly encountered during source localization of interictal spikes in people with intractable epilepsy. By comparing the results of Champagne to a conventional source localization technique, ECDss, we have shown its equivalency, and have also illustrated its use as a salvage technique in some scenarios where ECDss may fail. The results suggest that Champagne is a reliable alternative to manual ECDss dipole fitting for clinical application.

## Data Availability Statement

The raw data supporting the conclusions of this article will be made available by the authors, without undue reservation.

## Ethics Statement

The studies involving human participants were reviewed and approved by UCSF Committee on Human Research. Written informed consent to participate in this study was provided by the participants' legal guardian/next of kin. Written informed consent was obtained from the individual(s), and minor(s)' legal guardian/next of kin, for the publication of any potentially identifiable images or data included in this article.

## Author Contributions

CC, HK, JC, and SN contributed to the conception and design of the study. CC, JC, AF, DM, HK, and SN organized the database. CC, JC, AF, KS, HK, and SN performed the statistical analysis. CC, HK, and SN wrote the first draft of the manuscript. All authors contributed to manuscript revisions, read, and approved the submitted version.

## Conflict of Interest

KS was employed by company Signal Analysis Inc. The remaining authors declare that the research was conducted in the absence of any commercial or financial relationships that could be construed as a potential conflict of interest.

## Supplementary Material

Supplementary Table 1 Clinical characteristics of the 16 subjects. The first 14 described here appear in Table 1. Note that some patients were referred from outside institutions and thus their information was limited to that available at the time of the MEG scan. FCD = focal cortical dysplasia; Lt = Left; Rt = Right; temp = temporal; occip - occipital; ATL = anterior temporal lobectomy; MTS = mesial temporal sclerosis; ECoG = electrocorticography; ED = epileptiform discharge; PQ = posterior quadrant; GFPA = generalized paroxysmal fast activity; SEEG = stereo-EEG; hypo = hypometabolism; PVNH = periventricular nodular heterotopia; c/w = consistent with.
