# Supplementary material for: Clinical Validation of the Champagne Algorithm for Epilepsy Spike Localization
Source: Front Hum Neurosci. 2021 May 20;15:642819. doi: 10.3389/fnhum.2021.642819 (PMC8172809; doi:10.3389/fnhum.2021.642819)
Supplement: Supplementary Table 1 — Clinical characteristics of the 16 subjects. The first 14 described here appear in Table 1. Note that some patients were referred from outside institutions and thus their information was limited to that available at the time of the MEG scan. FCD = focal cortical dysplasia; Lt = Left; Rt = Right; temp = temporal; occip - occipital; ATL = anterior temporal lobectomy; MTS = mesial temporal sclerosis; ECoG = electrocorticography; ED = epileptiform discharge; PQ = posterior quadrant; GFPA = generalized paroxysmal fast activity; SEEG = stereo-EEG; hypo = hypometabolism; PVNH = periventricular nodular heterotopia; c/w = consistent with. [file Data_Sheet_1.PDF]

ID	Age	Duration of Epilepsy (years)	MR abnormality	Prior surgery?	Ictal EEG	PET	Ictal SPECT	presumed SOZ at time of referral for MEG
1	33	30	FCD inferior Lt temp and occipital lobes	no	Lt>Rt PQ	Hypo Lt>Rt mesial temp and over FCD	None	inferior Lt occipital and temp lobes
2	30	∼10	none	no	Rt midtemp with broad field and variable spread	Hypo Rt anterior temp	None	Rt mesial temp, posterior temp, or orbitofrontal
3	22	10	Rt occipital encephalomalacia	no	Rt PQ	None	only reduced uptake Rt occip (encephaloma-lacia)	Rt lateralized but poorly localized, with an atypical lesion
4	8	5	Normal	no	Rt temp, late onset relative to behavioral change	None	Rt temp and frontal	Rt temp
5	10		bilateral frontal and Rt atrial PVNH	no	GPFA	Normal		Presumed focal and symptomatic of PVNH but disparate structural and functional data; Rt central IED in sleep
6	16	1	Lt operculum and middle frontal gyrus signal change	no	Lt central	Hypo Lt frontotemp	Posterior Lt frontal; mesial, anterior, and lateral temp lobe	Lt frontal operculum/middle frontal gyrus vs temp lobe
7	20	15	Normal	no	Nonlocalized and nonlateralized; bifrontal Eds	None	None	likely frontal, but nonlateralized
8	19	12	Lt occipital resection cavity	lesionectomy of Lt occip FCD	Lt PQ	None	None	Lt PQ
9	23	18	Lt ATL resection cavity	ATL	No seizures captured	None	None	Lt temp, could include Heschl's/STG remnant and possibly insula
10	46	24	Lt inferior parietal FCD	no	Lt lateralized but poorly localized	Hypo Lt inferior parietal lobe	None	Lt inferior parietal FCD abutting postcentral gyrus or supplementary sensory cortex
11	39	24	No cortical abnormality; multiple bifrontal subcortical white matter abnormalities	no	Late Rt or poorly lateralized	Hypo Rt temp, Rt posterior inferior parietal lobule	late/failed	Rt hemisphere, temp versus inferior parietal/Rolandic
12	34	6	Lt temp-occipital resection cavity	topectomy of Lt temp-occip FCD	Lt occipital	Hypo Lt posterior temp-occipital	None	Lt PQ superior to resection margin
13	25	6	None	no	Rt hemisphere, probably temp	None	None	Rt centrotemp
14	18	3	none	no	GPFA, often shifted toward Rt hemisphere	Hypo Rt medial temp and infero-frontal	None	Rt frontal (inferior/polar) vs temp
15	14	9	none	no	Scalp poorly localized, ?Rt frontal; SEEG Lt insular > Lt frontal	normal	none	Lt hemisphere
16	2	2	Numerous tubers; post-laser ablation changes	Laser ablations of Lt frontal, parietal, temp, opercular tubers	Rt temporoparietal, then diffuse GFPA	Diffuse global hypo, additional hypo in anterior frontal lobes and Lt temp lobe c/w tubers	none	multifocal
